# Getting the Drift - Methyl Bromide Application and Adverse Birth Outcomes in an Agricultural Area

**DOI:** 10.1289/ehp.121-a198

**Published:** 2013-06-01

**Authors:** Julia R. Barrett

**Affiliations:** Julia R. Barrett, MS, ELS, a Madison, WI–based science writer and editor, has written for *EHP* since 1996. She is a member of the National Association of Science Writers and the Board of Editors in the Life Sciences.

Although occupational exposures to the pesticide methyl bromide are associated with numerous health problems,^1^ little is known about the potential effects to the general population of chronic low-level exposure. However, agricultural drift has been associated with prostate cancer in one study of adult males,^2^ and a handful of animal studies have suggested potential developmental toxicity, including reduced birth weight.^3^ A new study in *EHP* based on modeled methyl bromide exposure in a human population now reports associations consistent with experimental findings.^4^

Some uses of methyl bromide were phased out in 2005 under the Montreal Protocol on Substances that Deplete the Ozone Layer.^5^ However, the protocol permits several continued agricultural applications, or “critical uses,” due to a lack of technically and economically viable alternatives.^6^ One reason methyl bromide is so difficult to replace is that none of the alternatives have such broad activity at a cost growers can afford. There has been some research on combining other compounds, but so far there aren’t any cost-effective options that work as well.^7^ More than 1.75 million kg of the pesticide was applied in California in 2010.^8^

“Before we even started our research, we looked to see what was already known about this chemical, which is such an important pesticide in California agriculture,” says Kim Harley, study coauthor and associate director for health effects research at the Center for Environmental Research and Children’s Health, University of California, Berkeley. “There was very limited evidence for reproductive and developmental toxicity; there was almost nothing.” To learn more, Harley and her colleagues drew on data collected through the Center for the Health Assessment of Mothers and Children of Salinas (CHAMACOS) Study. This longitudinal cohort study began in 1999–2000 with the enrollment of 601 pregnant women living in the Salinas Valley, an agricultural area in Northern California, and was designed to assess children’s exposure to pesticides and other chemicals and potential related effects.

The researchers paired CHAMACOS Study data for a subset of 442 women with information from California’s Pesticide Use Reporting system, which logs detailed information about the timing, location, and amount of pesticide applications. Interviews with the women at baseline and during the course of their pregnancies provided demographic and health information. The Global Positioning System coordinates of their homes were meshed with data from the Pesticide Use Reporting system to reveal methyl bromide amounts applied within 1, 3, 5, and 8 km of each residence. Medical records included pregnancy duration and newborns’ length, weight, and head circumference.

**Figure:**
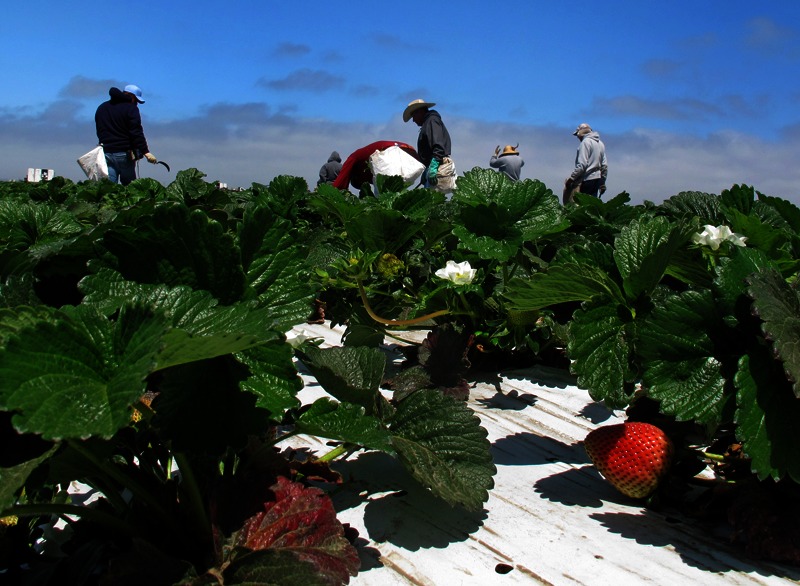
A strawberry field outside Salinas, California. Strawberries are one of several crops for which methyl bromide may still be used. © AP Photo/Gosia Wozniacka

The primary analysis focused on methyl bromide use within 5 km of a residence during each trimester of pregnancy. During the second trimester—a critical period of fetal growth—there were estimated decreases in average birth weight, length, and head circumference of 21.4 g, 0.16 cm, and 0.08 cm, respectively, for each 10-fold increase in methyl bromide use within 5 km.

Clinically, the estimated changes would be considered insignificant; however, the association suggests a downward shift in the overall distribution of growth variables with exposure during the second trimester. More highly exposed women had babies estimated to average 113.1 g (about 4 oz) lighter than those of unexposed mothers. By comparison, Harley says, smokers have babies that are about 150–250 g lighter than nonsmokers, “so this is not a trivial difference in birth weight that we’re seeing in the high-exposed groups versus the unexposed group.”

“The data are interesting,” says Lygia Budnik, a professor in the Division of Occupational Toxicology and Immunology, Institute for Occupational and Maritime Medicine, University Hospital Hamburg-Eppendorf, Germany. “The critical-use exemption aspect is very important.” Budnik emphasizes that in addition to use in agriculture, other uses of methyl bromide are still permitted, such as fumigation of freight containers. “We believe that more people are or were exposed than has been [reported], and many people might not be aware of the exposure and the resulting health problems,” she says.

The study’s strengths included detailed information about the women and their babies and about methyl bromide use near their homes. However, effects of other pesticides, especially chloropicrin, which is often combined with methyl bromide, could not be ruled out. Additionally, individuals’ actual exposure could not be assessed.

“That’s what’s really hard about methyl bromide—we don’t have a biomarker,” says Harley. Residential proximity to application sites provides a good estimate of exposure, but it only applies to exposure at home; exposure levels away from home are unknown. Budnik and her colleagues are currently working on a potential biomarker based on increased blood levels of mitochondrial DNA in individuals exposed to methyl bromide and other fumigants.^9^

## References

[1] Budnik LT Prostate cancer and toxicity from critical use exemptions of methyl bromide: environmental protection helps protect against human health risks. Environ Health 11 5 2012http//dx..org/. 10.1186/1476-069X-11-5 22284215 PMC3807750

[2] Cockburn M Prostate cancer and ambient pesticide exposure in agriculturally intensive areas in California. Am J Epidemiol 173 11 1280 1288 2011http//dx..org/. 10.1093/aje/kwr003 21447478 PMC3121318

[3] National Research Council, Board on Environmental Studies and Toxicology, Committee of Toxicology, Subcommittee for the Review of the Risk Assessment of Methyl Bromide. Methyl Bromide Risk Characterization in California. Washington, DC:National Academy Press (2000); http://www.nap.edu/catalog.php?record_id=9849

[4] Gemmill A Residential proximity to methyl bromide use and birth outcomes in an agricultural population in California. Environ Health Perspect 121 6 737 743 2013http//dx..org/. 10.1289/ehp.1205682 23603811 PMC3672911

[5] EPA. The Phaseout of Methyl Bromide [website]. Washington, DC:U.S. Environmental Protection Agency (updated 25 Jan 2013). Available: http://www.epa.gov/ozone/mbr/ [accessed 15 May 2013].

[6] EPA. Methyl Bromide: List of Critical Uses [website]. Washington, DC:U.S Environmental Protection Agency (updated 7 Dec 2012). Available: http://www.epa.gov/ozone/mbr/cueuses.html [accessed 15 May 2013]

[7] Starkey TE. The history and future of methyl bromide alternatives in the southern United States. USDA Forest Service Proceedings, RMRS-P-68 (2012). Available: http://www.fs.fed.us/rm/pubs/rmrs_p068/rmrs_p068_031_035.pdf [accessed 15 May 2013].

[8] DPR. The Top 100 Pesticides Used Pounds of Active Ingredients Statewide in 2010 (All Sites Combined). Sacramento, CA:California Department of Pesticide Regulation (2012). Available: http://goo.gl/RSMvW [accessed 15 May 2013]

[9] Budnik LT, Kloth S, Baur X, Preisser AM, Schwarzenbach H (2013). Circulating mitochondrial DNA as a biomarker linking environmental chemical exposure to early preclinical lesions.. PLoS ONE..

