# Erratum: “Getting the Drift: Methyl Bromide Application and Adverse Birth Outcomes in an Agricultural Area”

**DOI:** 10.1289/ehp.121-a211

**Published:** 2013-07-01

In the June 2013 News article “Getting the Drift: Methyl Bromide Application and Adverse Birth Outcomes in an Agricultural Area” [Environ Health Perspect 121:A198 (2013)], Lygia Budnik was identified as an assistant professor when she is, in fact, a full professor.  *EHP* regrets the error.

